# Optimizing inpatient access to oral contraceptives: A quality improvement approach in behavioral health

**DOI:** 10.1016/j.rcsop.2026.100703

**Published:** 2026-01-06

**Authors:** Rachel S. Pierce, Devon N. Crews, Benjamin J. Pierce, Courtney K. Wulffson

**Affiliations:** a Mayo Clinic Health System – Eau Claire, Eau Claire, United States of America; b College of Nursing, University of Wisconsin – Eau Claire, Eau Claire, United States of America

**Keywords:** Oral contraceptives, Inpatient behavioral health, Medication reconciliation, Quality improvement, Pharmacy workflow

## Abstract

**Background:**

Continuity of oral contraceptive use during hospitalization is critical for reproductive health, yet behavioral health settings often deprioritize this need. At a Midwestern community hospital, pre-admission oral contraceptives were frequently withheld due to absent workflows, inventory limitations, and provider uncertainty.

**Objective:**

The purpose of this quality improvement initiative was to design, implement, and evaluate a structured oral contraceptive procurement workflow to improve continuity of care and support reproductive autonomy for hospitalized behavioral health patients.

**Methods:**

A quality improvement initiative introduced a structured oral contraceptive procurement workflow in an adult inpatient behavioral health unit. Using Plan-Do-Study-Act cycles, the multidisciplinary team addressed medication reconciliation, pharmacy coordination, and provider education. Data were collected retrospectively (2022−2023) and prospectively (January–April 2025) for females aged 18–49 prescribed oral contraceptives prior to admission.

**Results:**

Oral contraceptive administration improved from 33 % (17/51) pre-intervention to 78 % (7/9) post-intervention (*p* = .023). Patients were seven times more likely to receive oral contraceptives after implementation, with a 95 % confidence interval ranging from 1.31 to 37.40. Missed doses persisted due to provider unawareness of workflow and outpatient pharmacy stock constraints.

**Conclusions:**

Implementing a standardized workflow significantly enhanced oral contraceptive continuity for behavioral health inpatients. This systems-based approach demonstrates the value of interdisciplinary collaboration and pharmacy integration in closing reproductive care gaps. Future priorities include sustainability, electronic medical record integration, and broader adoption to advance reproductive health equity.

## Highlights

• OC access rose from 33 % to 78 % after workflow implementation.
• Patients were 7× more likely to get OCs post-intervention (*p* = .023).
• A pharmacy workflow improved OC continuity in behavioral health inpatients.
• Multidisciplinary teamwork streamlined pharmacy coordination.
• The model is scalable and supports reproductive health equity.

Medication reconciliation is a critical patient safety practice during care transitions, aimed at reducing errors by reviewing a patient's complete pre-admission medication list.^1^ Recognized as a National Patient Safety Goal by the Joint Commission,^2^ it informs inpatient treatment decisions. Continuity of care is especially important for oral contraceptives (OCs), which provide therapeutic benefits beyond pregnancy prevention. Given the wide variety of OC formulations, inpatient workflows should support their continued use during hospitalization.^3^^,^^4^

## 1 Systemic disparities and behavioral health vulnerabilities

Women's health faces systemic disparities, particularly among those with behavioral health (BH) diagnoses. Barriers such as gender bias, restrictive policies, logistical challenges, and fragmented insurance limit access to reproductive care.^5^, ^6^, 7., 8 These inequities call for patient-centered quality improvement (QI) initiatives. This gap reflects broader reproductive and psychiatric healthcare disparities, where women with mental health conditions often experience compounded barriers to care. These include stigma, fragmented health systems, and underrepresentation in clinical guidelines, which collectively limit access to timely and appropriate contraceptive management. Inpatient BH settings are particularly vulnerable because reproductive health needs are frequently deprioritized, exacerbating inequities in continuity of care and reproductive autonomy.

### 1.1 Consequences of interrupted contraceptive care

Continuity of contraceptive care during hospitalization is often overlooked, especially in BH settings. Interruptions in OC use can lead to unintended pregnancies, hormonal imbalances, and exacerbation of conditions such as dysmenorrhea, endometriosis, and menstrual migraines.^9^, ^10^, ^11^ BH patients are particularly vulnerable due to compounded psychiatric and reproductive health risks.

### 1.2 Guidelines and current gaps in practice

Professional guidelines emphasize maintaining contraceptive continuity when clinically appropriate.12., 13, 14, 15. Despite these recommendations, inpatient workflows rarely prioritize OC continuation, often relying on patient-supplied medications or ad hoc pharmacy procurement.^4^ Standardized protocols and pharmacist involvement are critical for safe and timely administration.^16^

### 1.3 Clinical considerations, identified need, and purpose

While OCs may be withheld for venous thromboembolism (VTE) risk, most BH patients are ambulatory and young with few comorbidities.^17^ The Padua Prediction Score (PPS) guides VTE risk assessment; patients with PPS <4 are considered low risk and eligible for OC continuation.^18^ Prior to this initiative, lack of workflow, limited inventory, and provider uncertainty led to frequent OC discontinuation. A needs assessment revealed that only 33 % of eligible BH inpatients received OCs during hospitalization, despite low VTE risk, underscoring the need for a structured process. Compared to the broader 14–20 % baseline rate of essential medication disruption among BH inpatients, the observed 67 % missed oral contraceptive doses in this setting represents a markedly higher lapse, indicating that OC continuity is disproportionately compromised during BH hospitalization.^19^

The problem addressed in this study is the lack of a standardized workflow to ensure timely continuation of pre-admission oral contraceptives in behavioral health inpatient settings, resulting in significant care gaps. Therefore, the purpose of this QI initiative was to design, implement, and evaluate a structured OC procurement workflow to improve continuity of care and support reproductive autonomy for hospitalized BH patients.

## 2 Methods

### 2.1 Ethical considerations

The QI initiative was reviewed and approved by the Research Innovation Council at the Midwestern community hospital. The project was deemed exempt from full Institutional Review Board (IRB) review. All data were deidentified prior to analysis to protect patient confidentiality and securely stored using institutional protocols, including password protection, biometric authentication, and Okta Verify.

### 2.2 Design

This QI initiative used a pre–post intervention design to evaluate the implementation of an OC procurement workflow in an inpatient BH)setting. The project was guided by iterative Plan-Do-Study-Act (PDSA) cycles, allowing continuous refinement based on real-time feedback. Each cycle lasted one month, with tests of change conducted in February, March, and April 2025.

### 2.3 Setting and team

The initiative was conducted in a 304-bed Midwestern community hospital with a 24-bed adult inpatient BH unit serving medically stable adults admitted for acute psychiatric care. A multidisciplinary team—including pharmacists, BH providers, nursing students, and an obstetrician—collaborated with a local university. The principal investigator (PI), a nurse practitioner, coordinated stakeholder meetings and workflow development. Monthly planning sessions with pharmacy leadership and a pharmacy intern supported implementation.

### 2.4 Intervention

A structured workflow was implemented to ensure timely access to OCs for female BH inpatients. Key components included:

• OC availability list with National Drug Codes (NDCs) to identify equivalent brand and generic formulations.
• Coordination between inpatient and outpatient pharmacy teams for timely delivery.
• Specialist input from an obstetrician as needed.
• The workflow launched December 2, 2024, with communication distributed via email and hosted on OneDrive. Data collection occurred January 20–April 30, 2025, with monthly PDSA refinements.

### 2.5 Study population

Female BH inpatients aged 18–49 prescribed an OC prior to admission were included. Exclusion criteria: male patients, age outside range, no pre-admission OC, or Padua Prediction Score ≥ 4.

### 2.6 Outcome measures

The primary outcome measure was the proportion of eligible BH in-patients who received their pre-admission OCs during hospitalization. Secondary outcomes included the odds ratio of OC administration post-intervention compared to pre-intervention and the rate of missed doses.

### 2.7 Data collection and accuracy

Pre-intervention data (2022–2023) were collected via retrospective chart review and stored in Excel; post-intervention data (Jan–Apr 2025) were collected daily in REDCap by a pharmacy intern. Variables included OC type, source (patient-supplied, pharmacy-supplied, unavailable, refused), timing of first administration, admission/discharge dates, and length of stay.

Data completeness and accuracy were ensured through daily verification by the pharmacy intern and PI. Each record was cross-checked against the electronic health record (EHR) medication administration log and discharge summary. Discrepancies were resolved immediately, and missing data were flagged and corrected before analysis. Both retrospective and prospective datasets were reviewed for consistency in variable definitions.

### 2.8 Statistical analysis

Variation between pre- and post-intervention periods was assessed using Fisher's exact test to compare proportions of OC administration. Odds ratios with 95 % confidence intervals were calculated to quantify the magnitude of change. Descriptive statistics summarized missed dose rates and administration timing across both periods. A *p*-value <.05 was considered significant. Analyses were conducted using Python (v3.12.7) and SciPy.

## 3 Results

**Fig. B1:**
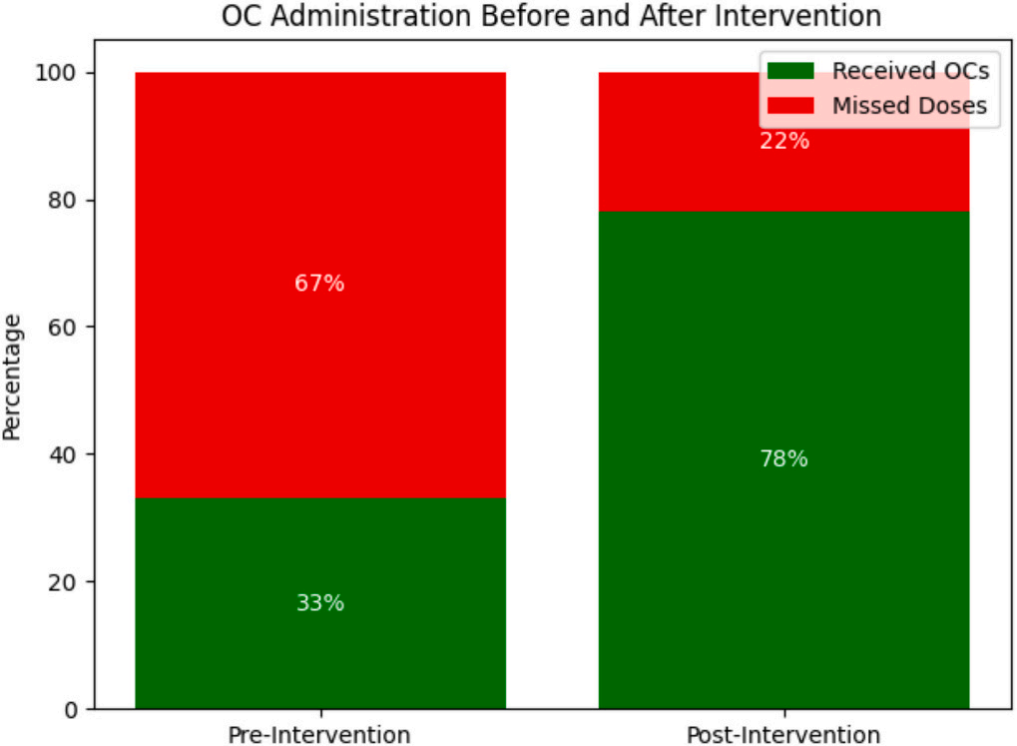
Rates before and after oral contraception procurement workflow intervention. *Note*. The pre-intervention involved a needs assessment that was conducted via retrospective chart review of females aged 18–49 admitted to the inpatient behavioral health (BH) unit between 2022 and 2023 prescribed a pre-admission oral contraceptive (OC). Following workflow implementation, OC administration increased from 33 % (*n* = 17, *N* = 51) to 78 % (*n* = 7, *N* = 9) of admissions. This improvement was statistically significant (*p* = .023), with patients being 7 times more likely to receive their pre-admission OCs post-implementation (95 % CI [1.31, 37.40]). In the post-implementation of the oral contraception procurement workflow, two OC administrations were missed—one due to the medication being unavailable at the onsite outpatient pharmacy, and the other due to an inpatient BH provider not continuing the patient's pre-admission OC order due to lack of awareness of workflow.

A retrospective chart review revealed that 67 % (*n* = 34, *N* = 51) of BH patients prescribed OCs during 2022–2023 experienced missed doses, highlighting a significant gap in medication continuity. Following implementation of the OC procurement workflow, continuity improved substantially. Between January and April 2025, 78 % (*n* = 7, *N* = 9) of eligible patients received their OCs compared to 33 % (*n* = 17) in the pre-intervention period (see Fig. B1). Missed doses decreased from 67 % to 22 %. Post-intervention, patients were seven times more likely to receive OCs (95 % CI [1.31, 37.40]; *p* = .23), indicating meaningful improvement in access and administration.

## 4 Discussion

### 4.1 Summary of key findings

This QI initiative addressed a critical gap in OC continuity for BH inpatients by implementing a structured procurement workflow. Leveraging outpatient pharmacy resources and multidisciplinary collaboration significantly improved medication reconciliation and access, reducing missed doses and supporting reproductive autonomy in a vulnerable population. These results demonstrate the effectiveness of systems-based interventions in closing care gaps.

### 4.2 Comparison to previous literature

This QI initiative aligns with existing literature underscoring the critical role of medication reconciliation and pharmacist involvement in enhancing continuity of care, particularly in BH settings. Prior studies have emphasized the importance of inpatient pharmacists in resolving medication-related issues during transitions^16^ and highlighted opportunities for BH providers to address reproductive health needs during hospitalization.^14^ However, this project offers a novel contribution by specifically targeting OC access within an inpatient BH unit—a setting where reproductive health is often overlooked. While national data reveal persistent barriers to contraceptive access,^6^^,^^8^ few interventions have focused on inpatient BH environments. The success of this initiative suggests that institutional workflows may be a modifiable barrier to contraceptive continuity. Tailored strategies, such as an OC availability list and collaboration with outpatient pharmacy services, addressed logistical challenges commonly cited in the literature.^4^ Moreover, the proactive involvement of a multidisciplinary team reflects best practices in collaborative care and likely contributed to the intervention's effectiveness.

### 4.3 Barriers in practice

Several noteworthy insights emerged during the implementation of the OC procurement workflow. First, despite targeted communication efforts, one BH provider was unaware of the new workflow. This underscored the importance of enhancing provider engagement through more comprehensive dissemination and training strategies. Second, although medication technicians or inpatient pharmacists were instructed to document the specific week of the patient's OC cycle, many patients were unable to recall this information. This reduced the ability to ensure accurate continuation of phasic contraceptive regimens. Outpatient pharmacy inventory constraints, particularly regarding OC formulations and therapeutic equivalents, can limit the overall reach of the intervention. Inventory decisions are informed by a cost-benefit analysis, with outpatient pharmacy staff conducting regular reviews to assess the need for adjustments. The OC availability list was positively received for enhancing clarity and workflow efficiency; however, its utility was limited when prescribed OCs were not stocked or when providers failed to reorder the OC. Additionally, on one occasion, the prescribed OC was not available in the outpatient pharmacy.

### 4.4 Limitations

This QI project has several limitations impacting its generalizability. A small post-intervention sample size and brief data collection period (Jan 20–Apr 30, 2025) limit statistical power and long-term assessment. Additionally, the study may be subject to bias due to manual data collection and reliance on a single site, which could limit the generalizability and reproducibility of the findings across other inpatient BH settings. The initiative remains active, but manual data tracking has ceased due to staffing changes. Reliance on individual champions and manual processes raises concerns about scalability and sustainability. While OC access improved, missed doses persisted due to ongoing systemic barriers, including provider unawareness and outpatient pharmacy stock limitations.

## 5 Conclusions

This QI initiative improved reproductive care continuity for female patients in an inpatient BH unit through a structured OC procurement workflow. As the unit expands to 32 beds, more patients may benefit from this approach. By addressing systems-level barriers, the project promoted reproductive autonomy and demonstrated that leveraging outpatient pharmacy resources can close care gaps without expanding inpatient inventory. Sustainability depends on institutional support, and the model is replicable across settings. Future research should explore a broader national patient care gap, electronic medical record (EMR)-integrated solutions, and long-term outcomes. At the project site, efforts continue to embed the workflow into standard procedures. This initiative aligns with the Joint Commission's National Patient Safety Goal on medication reconciliation by reducing missed OC doses during care transitions. The success of this initiative highlights the potential for institutional policy changes and integration into EMRs to standardize contraceptive continuity workflows, offering a scalable model that can be adapted across diverse inpatient settings to improve reproductive health equity.

## CRediT authorship contribution statement

**Rachel S. Pierce:** Writing – review & editing, Writing – original draft, Visualization, Validation, Supervision, Software, Resources, Project administration, Methodology, Investigation, Funding acquisition, Formal analysis, Data curation, Conceptualization. **Devon N. Crews:** Writing – review & editing, Writing – original draft. **Benjamin J. Pierce:** Writing – review & editing, Writing – original draft, Visualization, Validation, Supervision, Project administration, Methodology, Investigation, Formal analysis, Conceptualization. **Courtney K. Wulffson:** Writing – review & editing, Writing – original draft, Visualization, Validation, Supervision, Resources, Project administration, Methodology, Investigation, Formal analysis, Data curation, Conceptualization.

## Declaration of competing interest

Rachel S. Pierce reports relationships with University of Wisconsin-Eau Claire and Mayo Clinic Health System that includes employment. Benjamin J. Pierce and Courtney K. Wulffson report relationships with Mayo Clinic Health System that includes employment. The other author declares that they have no known competing financial interests or personal relationships that could have appeared to influence the work reported in this paper.

## Figures and Tables

**Fig. A1:**
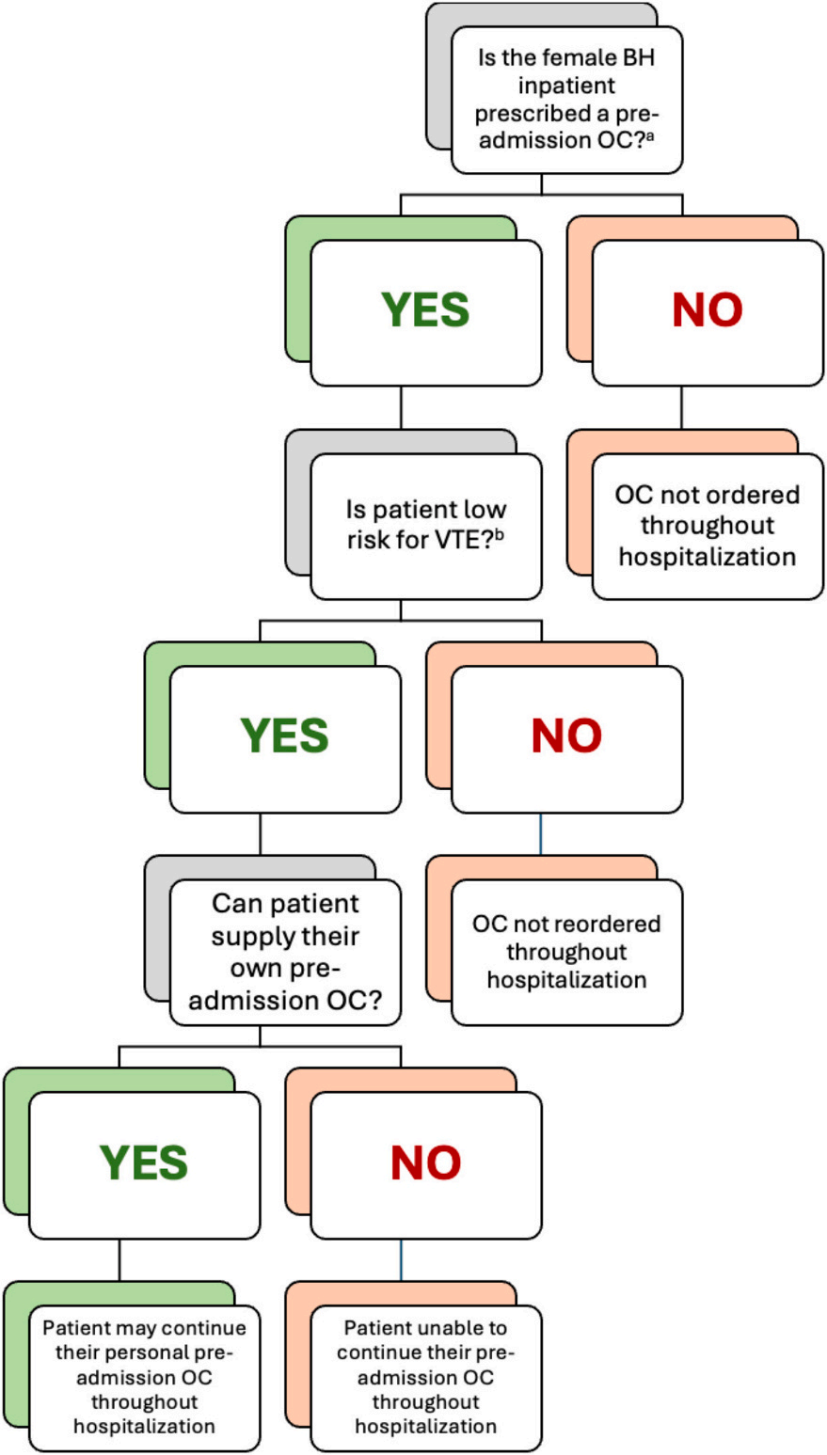
*Oral contraceptive ordering process: pre-workflow intervention. Note*. This workflow applies to behavioral health (BH) female inpatients prescribed an oral contraceptive (OC) prior to hospital admission. ^a^Pre-admission OC refers to any OC prescribed before the current hospitalization. ^b^BH providers determine that a pre-admission OC is appropriate to continue inpatient if patient is low risk of venous thromboembolism (VTE), such as a Padua Prediction Score (PPS) of less than 4.

**Fig. A2:**
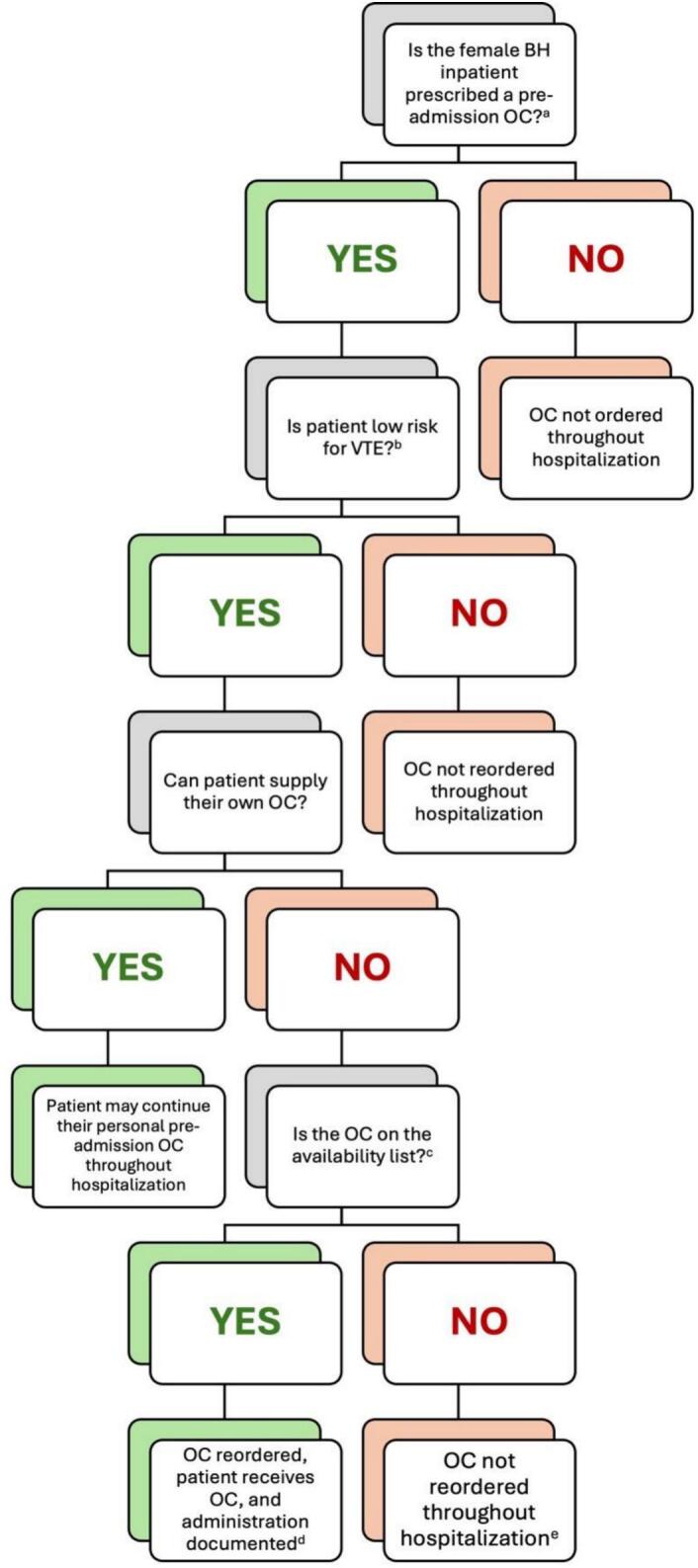
*Oral contraceptive ordering process: oral contraception procurement workflow (Simplified). Note*. This workflow applies to behavioral health (BH) female inpatients aged 18–49 prescribed an oral contraceptive (OC) prior to hospital admission. ^a^Pre-admission OC refers to any OC prescribed before the current hospitalization. ^b^BH providers determine that a pre-admission OC is appropriate to continue inpatient if patient is low risk of venous thromboembolism (VTE), such as a Padua Prediction Score (PPS) of less than 4. ^c^The OC availability list simplifies the classification of brand and generic OC products by highlighting therapeutic equivalence across products and which OCs are included in the onsite outpatient pharmacy's inventory. ^d^If appropriate, inpatient pharmacists order a monthly pack of the pre-admission OC from the onsite outpatient pharmacy to be delivered to the BH unit. In electronic medical record, BH nurse documents administration or reason for OC omission (e.g., refusal, unavailable, not ordered). ^e^If a pre-admission OC is not stocked in the outpatient pharmacy inventory, as indicated on the OC availability list, the OC is not reordered and is documented in the electronic medical record as unavailable.

## Data Availability

Data supporting the findings are available from the corresponding author upon reasonable request.

## Appendix C Supplementary data

Supplementary material Rates Before and After Oral Contraception Procurement Workflow Intervention. Supplementary material
