# Supplementary material for: Optimizing inpatient access to oral contraceptives: A quality improvement approach in behavioral health
Source: Explor Res Clin Soc Pharm. 2026 Jan 6;21:100703. doi: 10.1016/j.rcsop.2026.100703 (PMC12856171; doi:10.1016/j.rcsop.2026.100703)
Supplement: Supplementary material — Rates Before and After Oral Contraception Procurement Workflow Intervention. [file mmc1.docx]

**Optimizing Inpatient Access to Oral Contraceptives: A Quality Improvement Approach in Behavioral Health**

**Exploratory Research in Clinical and Social Pharmacy**

**01/02/2026**

We would like to thank the reviewers for their thorough and constructive reviews. The following table indicates our changes based on feedback.

| **Reviewer** | **Revision Suggested** | **Notes** |
| --- | --- | --- |
| Reviewer #1 | 1. Clearly state the **outcome measure** for the QI intervention. 2. In **Methods Section**, add details on:    a. How **data completeness and accuracy** were ensured.    b. How **variation between pre- and post-intervention periods** was assessed. 3. In the **Discussion section (first paragraph)**: Summarize key findings more succinctly, avoiding repeating previously presented results. | 1. On page 6 within the **Methods** section’s **Outcome Measures**, “The primary outcome measure was the proportion of eligible BH inpatients who received their pre-admission oral contraceptives during hospitalization. Secondary outcomes included the odds ratio of OC administration post-intervention compared to pre-intervention and the rate of missed doses.” was added. 2. a. On page 7 within the **Methods** section’s **Data Collection and Accuracy**, “Data completeness and accuracy were ensured through daily verification by the pharmacy intern and PI. Each record was cross-checked against the electronic health record (EHR) medication administration log and discharge summary. Discrepancies were resolved immediately, and missing data were flagged and corrected before analysis.” was added. 3. On page 8 within the **Discussion** section’s **Summary of Key Findings**, the paragraph was rewritten. Duplicate information from the **Results** section was removed and replaced as, “This quality improvement initiative addressed a critical gap in oral contraceptive continuity for BH inpatients by implementing a structured procurement workflow. Leveraging outpatient pharmacy resources and multidisciplinary collaboration significantly improved medication reconciliation and access, reducing missed doses and supporting reproductive autonomy in a vulnerable population. These results demonstrate the effectiveness of systems-based interventions in closing care gaps.” |
| Reviewer #2 | 1. Strengthen the **Introduction and Literature Review**:  - Expand coverage on:   a. Inpatient contraceptive continuation practices.   b. Prevalence and consequences of contraceptive disruptions.   c. Guidelines/recommendations from **ACOG**, **CDC/US MEC**, and **APA** on inpatient reproductive health management.  2. Explicitly state the **research problem** in a standalone sentence, e.g.: “The problem addressed in this study is the lack of a standardized workflow to ensure timely continuation of pre-admission oral contraceptives in BH inpatient settings, resulting in significant care gaps.” 3. Clarify how this problem fits within **broader reproductive and psychiatric healthcare disparities**. | 1. a. b. c. **Introduction** section was rewritten with additional information added to the **Systemic Disparities and Behavioral Health Vulnerabilities** section, **Consequences of Interrupted Contraceptive Care**, **Guidelines and Current Gaps in Practice** section, and **Clinical Considerations, Identified Need, and Purpose** section. Inpatient contraceptive continuation practices, prevalence and consequences of contraception disruption, and guidelines and recommendations on inpatient reproductive health management. ACOG, CDC, and APA guidelines included. 2. Research problem addressed in standalone sentence on page 4 Introduction in **Clinical Considerations, Identified Need, and Purpose** section as, “The problem addressed in this study is the lack of a standardized workflow to ensure timely continuation of pre-admission oral contraceptives in behavioral health inpatient settings, resulting in significant care gaps.” 3. On page 3 in the **Introduction’s** **Systemic Disparities and Behavioral Health Vulnerabilities** section, “This gap reflects broader reproductive and psychiatric healthcare disparities, where women with mental health conditions often experience compounded barriers to care. These include stigma, fragmented health systems, and underrepresentation in clinical guidelines, which collectively limit access to timely and appropriate contraceptive management. Inpatient BH settings are particularly vulnerable because reproductive health needs are frequently deprioritized, exacerbating inequities in continuity of care and reproductive autonomy” was added. |
| Additional Modifications | - Highlights document will also be submitted. - Abstract edited to 200 word-limit. - Minor formatting edit to a box-to-box line for Appendix A, Figure A1.   On 12/22/2025:   - A comma was added after e.g. note in Appendix A, Figure A2. - All retrieved dates for webpages in the reference list were updated to the current date of December 22, 2025. Reference numbers 2, 3, and 15. - Reference Number [9]: The hyperlink was edited to access the Up-to-Date website. - Reference Number [12]: The most recent American College of Obstetricians and Gynecologists “Access to Contraception” clinical guidance webpage was added. - Reference Number [13]: The Centers for Disease Control and Prevention article, “U.S. Medical Eligibility Criteria for Contraceptive Use, 2024”, was updated.   On 01/02/2026:   - **Abstract**: Objective subheading added following Background. The objective statement, was included as, "The purpose of this quality initiative was to design, implement, and evaluate a structured oral contraceptive procurement workflow to improve continuity of care and support reproductive autonomy for hospitalized behavioral health patients." - All acronyms in the abstract were removed, OC, BH, QI, and CI. - The abstract became longer with the removal of acronyms and the addition of the objective statement; therefore, Appendix A Figures 1 and 2 and Appendix B Figure B1 were resubmitted with updated page numbers. - On page 5, "BH" (behavioral health) was added to the purpose statement. - **References**: Changed the retrieved date to January 2, 2026 for references 2, 3, 9, 12, and 15. | |
